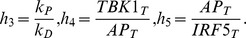# A Systems Model of Phosphorylation for Inflammatory Signaling Events

**DOI:** 10.1371/journal.pone.0110913

**Published:** 2014-10-21

**Authors:** Ildar I. Sadreev, Michael Z. Q. Chen, Gavin I. Welsh, Yoshinori Umezawa, Nikolay V. Kotov, Najl V. Valeyev

**Affiliations:** 1 Centre for Systems, Dynamics and Control, College of Engineering, Mathematics and Physical Sciences, University of Exeter, Harrison Building, Exeter, United Kingdom; 2 Department of Mechanical Engineering, The University of Hong Kong, Hong Kong, China; 3 Academic Renal Unit, School of Clinical Sciences, University of Bristol, Dorothy Hodgkin Building, Bristol, United Kingdom; 4 Department of Dermatology, The Jikei University School of Medicine, Minato-ku, Tokyo, Japan; 5 Biophysics and Bionics Lab, Institute of Physics, Kazan Federal University, Kazan, Russia; Yong Loo Lin School of Medicine, National University of Singapore, Singapore

## Abstract

Phosphorylation is a fundamental biochemical reaction that modulates protein activity in cells. While a single phosphorylation event is relatively easy to understand, multisite phosphorylation requires systems approaches for deeper elucidation of the underlying molecular mechanisms. In this paper we develop a mechanistic model for single- and multi-site phosphorylation. The proposed model is compared with previously reported studies. We compare the predictions of our model with experiments published in the literature in the context of inflammatory signaling events in order to provide a mechanistic description of the multisite phosphorylation-mediated regulation of Signal Transducer and Activator of Transcription 3 (STAT3) and Interferon Regulatory Factor 5 (IRF-5) proteins. The presented model makes crucial predictions for transcription factor phosphorylation events in the immune system. The model proposes potential mechanisms for T cell phenotype switching and production of cytokines. This study also provides a generic framework for the better understanding of a large number of multisite phosphorylation-regulated biochemical circuits.

## Introduction

Phosphorylation is the process by which a phosphate group is added to a protein. It leads to either activation or deactivation of a great number of proteins and represents a major building block for network regulation [1]. The addition of a phosphate group can occur either on a single site or on several sites, the latter is known as the multisite phosphorylation [2]. Multisite phosphorylation plays a key role in T and B cells activation. Aberrations in the phosphorylation mechanism are reported to give rise to autoimmune diseases [3]–[5].

Numerous studies designed to understand phosphorylation-mediated regulatory mechanisms have been reported recently. Early models employed Michaelis-Menten kinetics of the simplest phosphorylation reaction [6]. This model was expanded to include multiple phosphorylation reactions and demonstrated how these could enhance the sensitivity of biochemical systems [7]. It was also reported that such a system represents a switch when the total concentration of the substrate protein significantly exceeds the concentration of the enzyme [8].

The classical models assume that it is possible to ignore the concentrations of the Michaelis complexes in those cases where the total concentration of protein substrate significantly exceeds the concentrations of the kinase and the phosphatase. This approach was used as a basis in many biochemical networks with phosphorylation-dephosphorylation reactions [9]–[11] and was later extended to multisite phosphorylation [12], [13].

The proportion of maximally phosphorylated substrate as a function of the kinase and phosphatase activities was recently determined to show that steeper switch-like regulation is due to increasing of number of phosphorylation sites [14]. Moreover, the presence of multiple phosphorylation sites enhances the probability of bistable behavior of the system when tethered with scaffold proteins [15]. The properties of a bistable switch have recently been investigated to conclude that the mechanism must be distributive to generate multiple steady states and that bistability is more likely with a large number of phosphorylation sites. The phenomenon of ultrasensitivity has also been reported to increase linearly with the number of phosphorylated sites [16].

Phosphorylation plays a critical role in the regulation of the immune system. However, there is a clear gap in the mechanistic understanding of the role of multisite phosphorylation in this process. Phosphorylation governs protein signaling via Signal Transducers and Activators of Transcription (STAT) proteins [17]–[19].

The STAT proteins are critical for many fundamental cellular processes such as proliferation, differentiation, cell growth and survival [20]. They operate in the ubiquitous JAK/STAT pathway. There are seven mammalian STAT proteins each with a specific role in the immune system. A considerable amount of experimental evidence shows that dysfunction in the JAK/STAT signalling mechanisms leads to inflammatory diseases [21]–[26].

The STAT proteins are activated by phosphorylation of their C-terminal transactivation domain (CTD) by Januse Kinases (JAKs) at Tyr701 for STAT1 in response to type II interferons [27] and Tyr705 for STAT3 in response to Interleukin 6 or 10 [28], [29]. Phosphorylation at Tyr705 leads to the dimerization [30] and regulates the activation of STAT3 [31]–[33]. There are three classes of STAT negative regulators: Suppressors of Cytokine Signaling (SOCS), Protein Inhibitors of Activated STATs (PIAS) and the simplest class Protein Tyrosine Phosphatases (PTPs), for instance SHP-1, which reverses the activity of the JAKs [18], [34].

Interferon Regulatory Factor 5 (IRF-5) is a latent transcription factor involved in autoimmunity [35]. IRF-5 is known to contain six phosphorylation sites: Thr10, Ser158, Ser309, Ser317, Ser451 and Ser462, but only the last two have so far been shown to be functional [36], [37].

Several models for STAT3 and IRF-5 phosphorylation as part of larger models have been published recently. A classical approach for the phosphorylation of STAT3 by JAK has been employed in [38]. Another report proposed sigmoidal Hill functions for phosphorylation of STAT3 [39]. An explicit mathematical model for IRF-5 phosphorylation is not currently available, but the phosphorylation of IRF-3 as part of the TLR4 pathway has been considered [40].

The cells that differentiate in the thymus and are involved in cell mediated immunity are known as T cells. They circulate in the lymphoid organs and the blood in the form of naive T cells, which have not been in contact with antigens yet. After the interaction with the antigen the naive CD4+ T cells are activated and can differentiate into the specific T cell phenotypes, namely T helper 1 (Th1), Th17 and regulatory T cells (Tregs). Each of these phenotypes has its own function in the regulation of the immune response and a specific cytokine signature. Th1 and Th17 cells play a critical role in the regulation of the activity of the immune response and inflammation. Tregs are known for their anti-inflammatory properties and for maintaining the immune tolerance. Th1 cells are defined by expressing IFN-γ, Th17 cells by IL-17 and Tregs by IL-10 [41], [42]. The specific phenotype is induced by the production of the specific cytokines. For example Th1 is induced by IL-12, Th17 by IL-6 and Tregs by TGF-β. These cytokines activate specific transcription factors, involved in the differentiation of the T cell subsets [43]. Thus, the differentiation of T cells is a complicated process involving a complex scheme of regulation by cytokines and transcription factors. In this work we focus on two of them, IRF-5 and STAT3 assuming the underlying mechanism of the activation of other IRFs and STATs is similar to the one we propose here.

In this study a new model for multisite phosphorylation has been developed. The model has been compared with previously reported models [12]–[16] in the context of experimental data for intracellular signaling of the inflammatory circuits [44], [45]. Specifically we applied the model to investigate the underlying molecular mechanisms of STAT3 and IRF-5 signaling pathways. We employed the developed model to investigate the parametric sensitivity of the inflammatory circuits in response to various inflammatory co-stimuli. This analysis was performed in comparison with the previously proposed mathematical models for multisite phosphorylation [12]–[16]. We show that the applicability of earlier models [12]–[16] is limited with respect to understanding signaling in the immune system.

## Results

### A new model for multisite phosphorylation

In this study, we developed a new mathematical model for multisite phosphorylation signaling. The model predicts probabilities for a protein to be phosphorylated at various phosphorylation sites as a function of the kinase activity. The newly developed and previously reported models were compared with the experimental data for the transcriptional regulation of the STAT and IRF-5 proteins.

**Figure 1:**
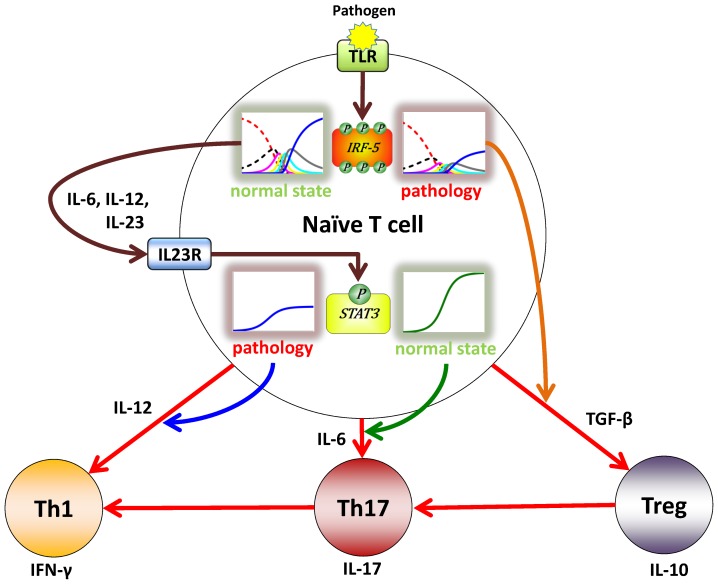
A schematic diagram for the dependence of T cell differentiation on intracellular phosphorylation signaling. A vast amount of experimental evidence suggests that T cell phenotypes strongly depend on the intracellular phosphorylation signaling mechanisms [44]–[50]. Environmental factors, genetic mutations, cellular and intracellular factors influence the underlying phosphorylation mechanics. The cartoon summarizes possible differential responses of TLR downstream phosphorylation signaling events to pathogens leading to the distinct polarization of naive T cells into three distinct phenotypes Th17, Th1 and Treg. According to this model activation or interplay of phosphorylation pathways is responsible for selective differentiation as well as for T cell phenotype switching. The model suggests that the cell plasticity observed under pathological conditions can be due to altered intracellular phosphorylation patterns, which are, in turn, dependent on the extracellular cytokine environment.

It has been shown that IRF-5 contributes to the polarization and plasticity of macrophages [44]. Pathogens such as bacteria and viruses cause the activation of the Toll-Like Receptors (TLRs). This signaling leads to the activation of IRF-5 [46] and the production of pro-inflammatory interleukins IL-6, IL-12 and IL-23 [44], [47]. These cytokines are able to activate STAT3 and result in the Th17 differentiation [45], [48]. Treg cells then can switch to Th17 cells [49] which in turn then can switch to Th1 subpopulation [50]. In this study we propose a model based on the reported experimental data according to which different types of signals result in different types of immune response. Figure 1 schematically represents an experimental-data based model for the role of IRF-5 and STAT3 in T cell fate determination. Due to the highly competitive nature of the pathways of the scheme any disturbances in the mechanism may lead to the enhancement of the role of other cytokines and formation of different types of T cells. Such perturbations are schematically shown by the patterns on right for IRF-5 and on left for STAT3 and highlighted by the red glow while the normal regulation is highlighted by green. Since the classical approach [6] offers rather limited representation of the underlying mechanism, it potentially leads to somewhat incorrect interpretation of the experimental data. The proposed model offers more physiologically accurate description of the role of multisite phosphorylation regulation of the T cell differentiation.

**Figure 2:**
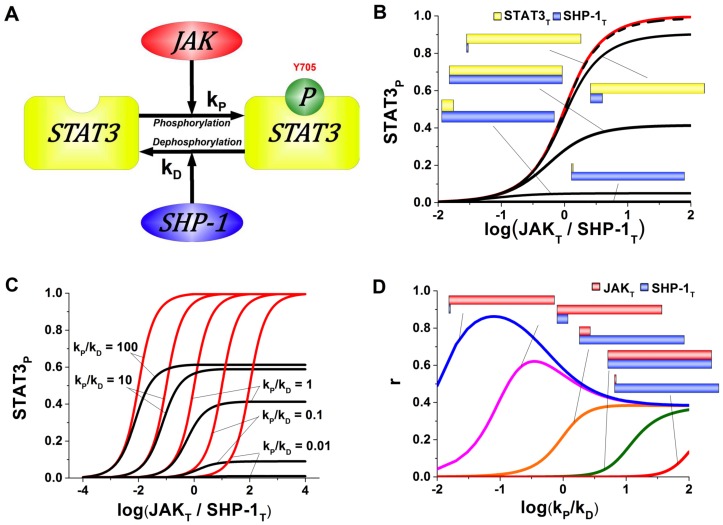
Model predictions for the concentration of STAT3_p_ phosphorylated by JAK and dephosphorylated by SHP-1. Investigation of the dependence of STAT3 phosphorylation on the relative activities of JAK and SHP. (A) Cartoon diagram of STAT3 phosphorylation and dephosphorylation by JAK and SHP-1, respectively. (B) A comparative analysis of the proposed and applications of the previously published models for STAT3 phosphorylation. Ratios of JAK and SHP-1 are found to be critical for STAT3 phosphorylation response and the differences between the model predictions. The red line shows the predictions by [6] whereas the black line offers predictions from the presented model. The STAT3 phosphorylation predictions coincide when STAT3 significantly exceeds SHP-1 concentration. (C) The effects of phosphorylation and dephosphorylation rates are studied on the proposed (black line) and previously reported (red line) models [6]. We found that our model predicts the modulation of phosphorylated STAT as opposed to the prediction of STAT3 phosphorylation rate offered by [6]. (D) The comparison between the proposed and the previously model [6] is shown as a function of phosphorylation/dephosphorylation rates for various ratios of JAK and SHP-1. This analysis clearly demonstrates the differences in STAT3 phosphorylation predictions due to the underlying assumptions employed in the models.

Figure 2 shows the model predictions for the normalized steady-state activities of the phosphorylated STAT3 proteins denoted by STAT3_p_ (Figure 2A) and highlights the differences of the predictions for the phosphorylated STATs between the biochemically detailed model and previous simplified model [6]. The model proposed in this paper is consistent with the experimental observations of the phosphorylation events [44]–[48], [50] summarized in Figure 1 and predicts the mechanisms for the role of SHP-1 in modulation of the signal transduction via STATs [44], [45]. At the same time, some of the predictions of the presented and earlier models partially coincide for those cases when the JAK_T_ kinase and the SHP-1_T_ phosphatase concentrations are significantly smaller than the total concentrations of STAT proteins (STAT3_T_). However the model predictions differ when the corresponding concentrations are similar.

The figures show that the system may operate in a switch-like manner with an increasing concentration of JAK_T_ kinase, which leads to the ultrasensitivity that is characterized by the concentrations of STAT3_p_ being more sensitive to change in stimulus than would be expected from a Michaelis-Menten response [6]. This all-or-none characteristic of the response is observable not only in this particular system but in other cell systems such as Xenopus Oocyte extracts [51], [52], the glycogen cascade system [53], and ligand-receptor complexes [54].

Figure 2B shows the normalized concentration of phosphorylated STAT3 as a function of the kinase to phosphatase ratio (total amounts of JAK and SHP-1, respectively). The model predicts that the degree of STAT3_p_ activity depends on the ratio of the total STAT3 and SHP-1 concentrations. This prediction differs from the previous study [6], where the derived formula for phosphorylation could not reproduce this effect under certain physiological conditions. At the same time, the model predictions virtually coincide with the predictions from [6] if the concentration of SHP-1_T_ is significantly smaller than STAT3_T_. However, our model offers significantly different predictions for comparable or higher phosphatase concentrations than STATs, consistent with the T cell phenotype dependence on intracellular phosphorylation signaling summarized on Figure 1 [44]–[48], [50]. The proposed results are significant, as the relative ratio of STAT3 and SHP-1 has been shown to be critical in T cell breast lymphoma and Hepatocellular Carcinoma pathologies [55], [56].

Our calculations suggest that if the STAT3_T_ and SHP-1_T_ concentrations are comparable, the phosphorylated STAT3 species (STAT3_p_) increase as a function of the ratio of the forward phosphorylation reaction rate, 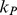, to the forward dephosphorylation rate, 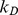 (Figure 2C). The introduction of the kinase-protein and/or phosphatase-protein complexes enables additional regulatory capacity of the STAT signaling events. While the simplified model predicts earlier or later STAT activation on the relative kinase/phosphatase activity scale, the new model suggests additional regulatory steps taking place via modulation of the total amplitude. This result is critical from the immunological point of view, as it explains some aspects of the functional plasticity of T cell phenotypes. According to our model, T cell populations may undergo different transcriptional activation events in response to the same stimuli due to different kinase and phosphatase activity levels. Furthermore, since the kinase and phosphatase activities are subject to short and long term modulation, this gives rise to possible phenotype switching. It is critical to highlight that these effects can be described using the proposed detailed phosphorylation reaction model only. The range of the tested parameters suggests that the differences between this and the other models are due to the structure of the model rather than the parameters (Figure 2C and Figure 2D).

Our analysis suggests that the STAT3 phosphorylation system with switch-like characteristics depends on the parameters of the model. The approach proposed by Goldbeter and Koshland [6] is only applicable for limited physiological conditions when the concentration of JAK_T_ and SHP-1_T_ are significantly smaller than that of STAT3_T_. While these situations can occur in nature, most living cells exhibit comparable concentrations of enzymes and their substrates. Therefore, the physiological range of applications considered in [6] is rather limited and all other phosphorylation events require the extended analysis described in this study.

### Application of the multisite phosphorylation model to the IRF-5 regulation

**Figure 3:**
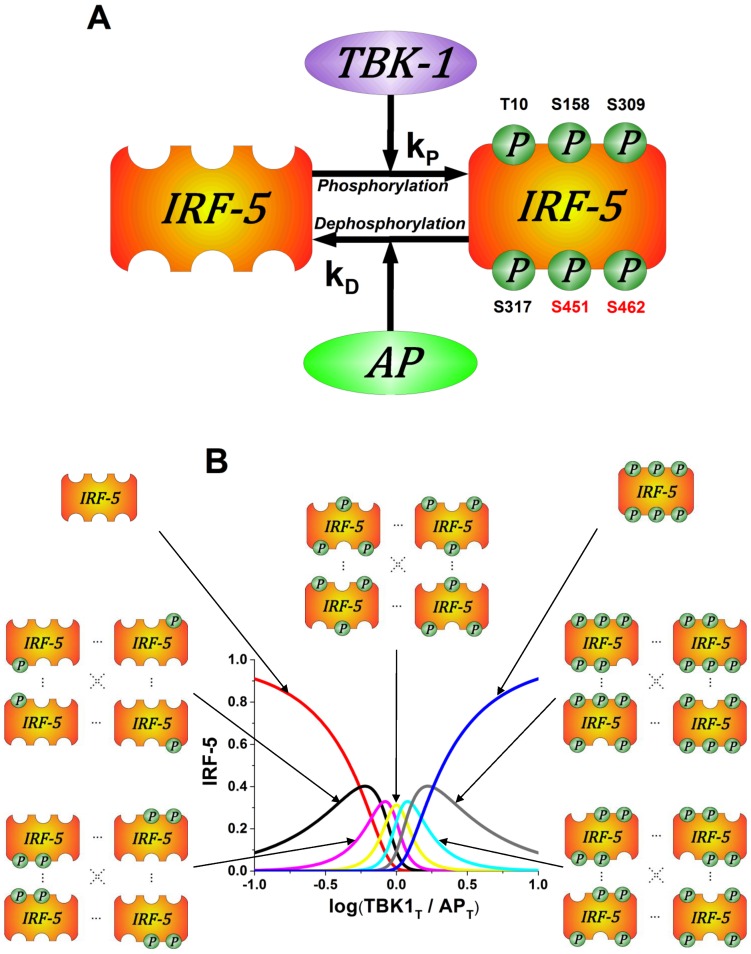
Multisite phosphorylation enables switching between multiple T cell phenotypes. (A) Schematic diagram of IRF-5 phosphorylation and dephosphorylation by TBK-1 and AP, respectively, represents one of many intracellular multiphosphorylation examples observed in the immune system. Experimental evidence suggests that proteins phosphorylated at different phosphorylation sites may have selective activity [37], [61] and give rise to distinct T cell populations [62]. (B) Computational model predictions for distribution of IRF-5 phosphorylated species. Extracellular environment is hypothesized ratio causing the distribution of IRF-5 phosphorylated species: one site phosphorylated (black), two (magenta), three (yellow), four (cyan), five (grey) and six (blue). According to the proposed model extracellular environment can actively change the ratio of IRF-5 phosphorylated species and thereby contribute to the mechanism of T cell plasticity by modulating the numbers of T cell phenotypes.

We next investigated multisite phosphorylation reactions in other inflammatory signalling pathways and studied the activation of IRF-5 as an example. IRF-5 is phosphorylated by the TBK-1 kinase and dephosphorylated by Alkaline Phosphatase (AP) (Figure 3A) [36], [37], [57]. Figure 3B shows the model predictions for the distribution of the phosphorylated IRF-5 species. It can be seen from the graph that the shape of the non- and fully-phosphorylated protein species qualitatively coincides with the case of the single-site phosphorylation reaction. However, the steepness of the phosphorylation response is significantly higher in the multisite phosphorylation reaction. The model predicts the bell-shaped dependence for the intermediate species and provides a clear explanation as to how receptor-mediated activatory events can be followed by inhibition in response to the same signal. The model predictions for the multisite phosphorylation reactions obtained in this study are consistent with previously reported results [58]. The model predictions in the form of bell-shaped curves for the intermediate phosphorylated protein species are consistent with the experimental data which suggests that IRF-5 requires phosphorylation of at least two sites for activation [36], [37].

We compared our model predictions with the previously reported method of Goldbeter and Koshland [6]. There are two key biochemical factors that may significantly vary in living cells and thereby affect the signaling properties: the ratio of total protein to kinase and phosphatase concentrations and the rates of phosphorylation, 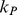, and dephosphorylation, 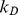, reactions. For simplicity, we did not vary the phosphatase concentration and changed the kinase activity only.

**Figure 4:**
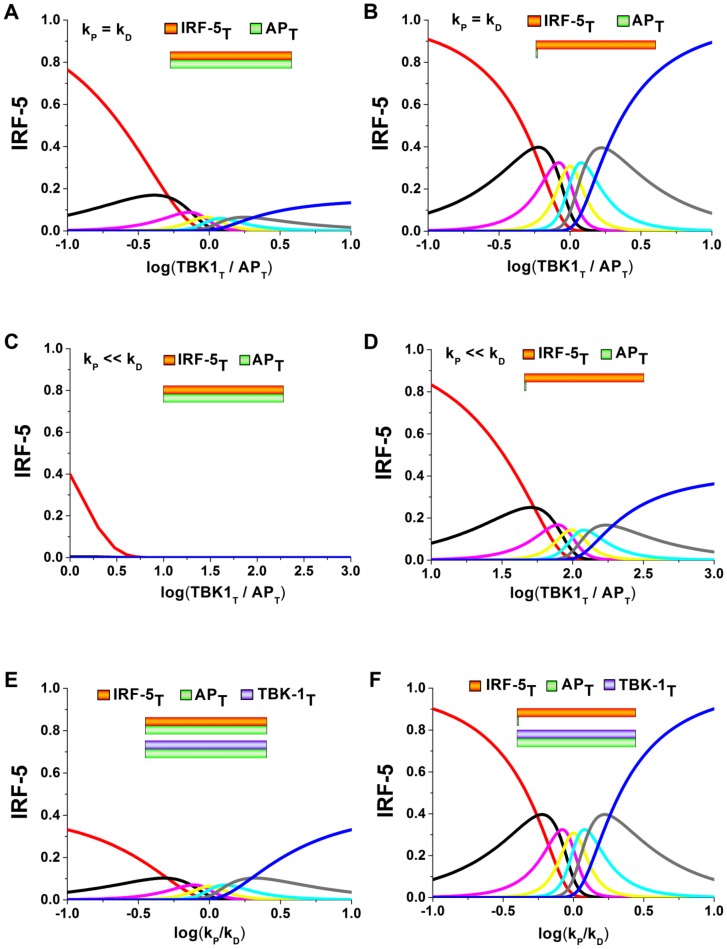
Theoretical investigation of the regulation of IRF-5 multisite phosphorylation. The distribution of IRF-5 species was investigated as a function of kinase to phosphatase (TBK to AP) ratio for comparable IRF-5 and AP concentrations (A), IRF-5 significantly exceeds AP (B). Similar analysis was also performed when the phosphorylation rate was significantly lower than dephosphorylation rate and comparable IRF-5 and AP concentrations (C), IRF-5 significantly exceeds AP (D). The effects of changes in the phosphorylation to dephosphorylation ratio on the IRF-5 species were also investigated with comparable IRF-5 and AP concentrations (E), IRF-5 significantly exceeds AP (F). The presented analyses clearly show that the phosphorylation/dephosphorylation parameters, modulated via extracellular cytokines have prominent impact on the distribution of phosphorylated species. Therefore, physiological or pathological alterations of these parameters represent the multisite phosphorylation-mediated mechanism of T cell plasticity.

Our analysis shows that alterations of phosphorylation rates and total IRF-5 to AP ratios do not have any impact on the model in [6]. Figure 4 shows the range of the model prediction for the described variation of parameters. It can be seen from the Figure 4 that for comparable phosphorylation to dephosphorylation rates, the non-phosphorylated form of IRF-5 appears to be dominant (Figure 4A). At the same time, the predictions from this model coincide with the predictions from [6], both when the phosphorylation and dephosphorylation rates are of the same order (Figure 4B) or different (Figure 4F), but only when the total amount of the IRF-5 concentration exceeds AP. We next decreased the phosphorylation rate (Figure 4C and Figure 4D) and investigated the case of comparable IRF-5 and AP concentrations (Figure 4C) compared with the case where the total concentration of IRF-5 is much larger (Figure 4D). The model predicts that most of the potential multisite protein species would remain unphosphorylated in the former case (Figure 4C), and would have a distribution very similar to the case of comparable phosphorylation rates and concentration shown in Figure 4A (Figure 4D). Our model predicts that the overall amount of all the phosphorylated protein species decreases significantly when the total TBK-1 concentration is comparable with the total IRF-5 concentration (Figure 4E).

Our results suggest that the selective activity of the multisite phosphoprotein-mediated response is regulated by the ratio of the total amounts of the protein to phosphatase and by the relative rates of the phosphorylation-dephosphorylation reactions. These effects have not been observed in previously reported mathematical models of phosphorylation [2], [6], [8], [11], [59]. The comparison of our analysis with experimental data [44]–[48], [50] summarized on the Figure 1 allows us to conclude that the multisite phosphorylation reactions enable diverse cellular activatory profiles in response to slight variations in the extracellular signal.

## Discussion

In this study we propose a new model for multisite phosphorylation with applications to intracellular signaling in the immune system. The model extends previous models for activation of proteins by single- [6]–[11] and multi-site phosphorylation [12], [14], [15], [58], [60]. The model offers more accurate predictions for phosphorylation-mediated regulation. This finding is obtained by the comparison with previously published models and experimental information for intracellular inflammatory circuits.

The proposed model has been applied to the STAT3 signaling circuit and compared with one of the previously published models [6]. Our analysis suggests that the Goldbeter and Koshland model [6] can be used only in the case when the total concentrations of JAK and SHP-1 are much lower in comparison with the total concentration of STAT3. However, in real systems the concentrations of kinases and their substrates are comparable [8]. Therefore, the concentrations of intermediate phosphorylation complexes cannot be ignored. Our model offers more accurate predictions for STAT3 phosphorylation. Since similar stimuli may lead to different transcriptional activation events and T cell phenotype switching, the results obtained in this study allow us to demonstrate that the lack of an accurate phosphorylation magnitude predictions may lead to misleading interpretation of the STAT-mediated T cell fate determination.

We show here that IRF-5 is activated in a switch-like manner (Figure 3 and Figure 4), which leads to the production of inflammatory cytokines such as IL-12 and IL-23 [44]. Our model suggests that this switch is highly dependent on the parameters of the system, particularly the ratio of the total AP to IRF-5 concentrations and phosphorylation/dephosphorylation reaction rates. Several autoimmune inflammatory diseases including Systemic Lupus Erythematosus (SLE) are due to the aberrations in the mechanism of IRF-5 activation. A more accurate description of the regulatory role of IRF-5 gives a clearer insight into a number of inflammatory diseases.

## Conclusions

This work introduces universal mechanisms for single and multisite phosphorylation and proposes an accurate model for phosphorylation-mediated regulation. The analysis reveals the physiological conditions under which the model coincides and differs from the classical models. This approach can have applications in a variety of molecular systems where the information is transmitted through a phosphorylation mechanism. The predictions of the model applied to the STAT3 and IRF-5 regulatory circuits has a broad impact to Systems Immunology and may increase our understanding of the mechanisms of inflammatory diseases.

## Materials and Methods

### Single site phosphorylation

Here we consider a general mechanism of phosphorylation of protein 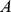 by the kinase 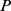 and dephosphorylation by phosphatase 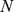.

The reactions can be represented as follows:
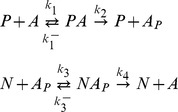



We introduce the following notation: 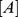, 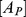 – the concentrations of the non-phosphorylated and phosphorylated protein respectively, 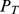, 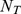, 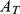 – the total concentrations of the proteins in an active form, 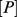, 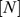
– the concentrations of free proteins, 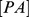, 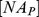 – the concentrations of kinase-protein and phosphatase-protein complexes, respectively. The kinetic equations of this molecular system are given by:
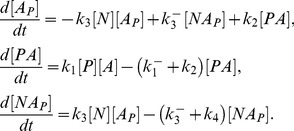
(1)


The conservation equations for the elements involved in the above reactions are as follows:
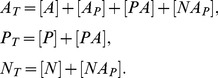
(2)


The steady-state solutions of 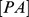 and 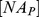 can be written as follows:
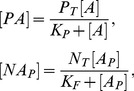
(3)where 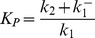 and 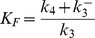 are the Michaelis constants for the phosphorylation-dephosphorylation reactions.

The rate of change of 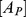 can be written as a function of 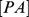 and 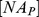:
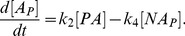
(4)


From Equations (2), (3) and (4) it can be written:
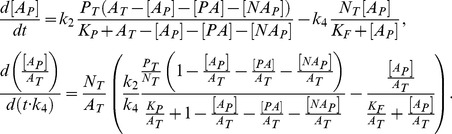
(5)


The non-dimensional form of the Equations (4) and (5) can be written as follows:
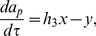
(6)

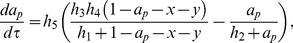
(7)where
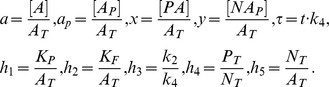



According to the above notation, Equations (3) in a non-dimension form are given by:
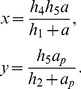
(8)


We can therefore rewrite the law of mass conservation for 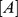 as follows:
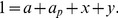
(9)


In [6], the steady state solution of 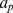 was found for 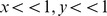. The sufficient condition to satisfy 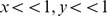 is 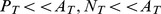, which means 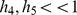. This implies that the concentrations of these complexes are negligible comparing to 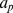 and the solution can be written as follows:
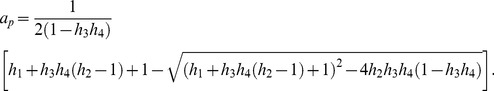
(10)


In general, conditions 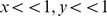 are not satisfied. We find an accurate solution of Equation (7). From Equations (8) we obtain:
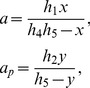
(11)where 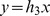 according to the steady state of Equation (6) when 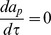.

Substituting Equations (11) into Equation (7) for steady state, we obtain:
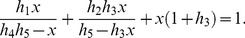
(12)


The range of 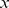 is limited and determined from the conservation equations:
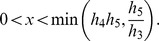



It can be shown that Equation (12) has one real root and two complex conjugate roots. Equation (12) can be written as follows:
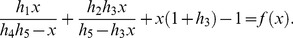
(13)


For any real positive values of the parameter 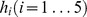, 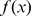 is a monotonically increasing function (it is continuous on the domain 
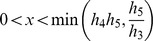
 and its derivative is positive for any value of 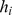), it has one intersection point with the horizontal axis, where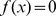, which means that there is only one real root of Equation (13).

We find the real root of Equation (13). Equation (12) can be transformed to the following equation:
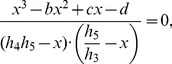
(14)where
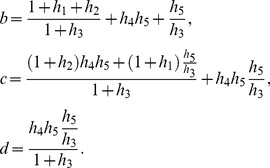
(15)


Since the domain is 
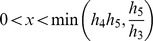
, the roots of the numerator in Equation (14) are equal to the roots of Equation (14), thus the latter can be simplified as:
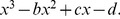
(16)


To find the roots of Equation (16) we used Vieta's formulas. Based on the fact that this equation has one real root 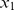 and two complex conjugate roots 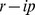 and 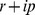, it can be written as follows:
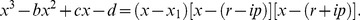
(17)


Thus there is a system of equations for 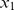, 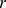 and 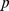:
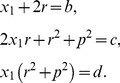
(18)


To simplify the above system of equations, we use the following parameters:
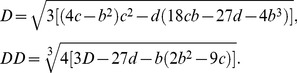
(19)


Solving the system described by Equation (18), the real solution of Equation (13) can be obtained:
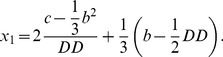
(20)


Thus, a steady-state solution for 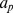 in a general form is:
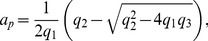
(21)where
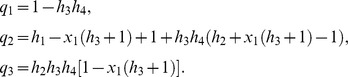



From Equations (11), parameters 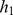 and 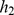 can be obtained:
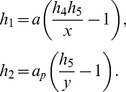
(22)


Since 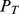, 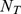, 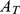 are known and 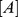, 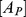, 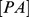, 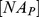 can be measured experimentally, we can find the Michaelis constants 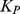, 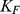:
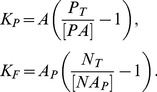
(23)


### Multisite phosphorylation

In this section we consider a system with 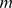 independent phosphorylation sites. The ODEs and the equations describing the final formula for the concentration of the protein phosphorylated at one single site are the same as in the last section, but the conservation equation for the total amount of protein differs from Equations (2). Instead of 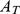, the total amount of protein is 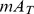 as the molecule has 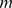 phosphorylation sites:
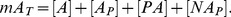
(24)


In this case, the normalized parameters are written as follows:
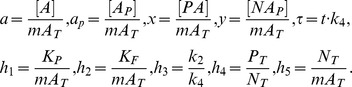



When 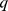 out of 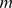 sites are phosphorylated it can be assumed that these events are independent and the sites are identical. Thus, the multisite phosphorylation is a combinatorial problem and can be considered in terms of the probabilities of the protein to be phosphorylated at distinct sites. Hence, the concentration of the protein phosphorylated at 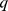 out of 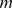 phosphorylation sites is proportional to the sum of all molecule combinations:
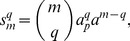
(25)where 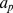 is the probability of the protein to be phosphorylated at a single site, 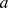 is the probability of the protein to be non-phosphorylated at a single site and 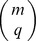 is a binomial coefficient.

Equation (25) can be written in detail as follows:
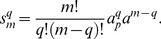
(26)


### STAT3 phosphorylation

STAT3 can form a dimer and be activated when it is phosphorylated at one site by JAK and dephosphorylated by SHP-1. Thus, Equations (10) and (21) can be used to denote the STAT3 concentration. The following notation is used in our model: 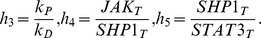



### IRF-5 phosphorylation

Here we consider phosphorylation of Interferon regulatory factor 5 (IRF5). Our model assumes that the molecule contains 6 independent phosphorylation sites. IRF-5 can be phosphorylated by TBK-1 kinase and dephosphorylated by Alkaline Phosphatase. In this case we use Equation (26), assuming